# Methods in Experimental Work Break Research: A Scoping Review

**DOI:** 10.3390/ijerph16203844

**Published:** 2019-10-11

**Authors:** André Scholz, Johannes Wendsche, Argang Ghadiri, Usha Singh, Theo Peters, Stefan Schneider

**Affiliations:** 1 Department of Management Science, Bonn-Rhein-Sieg University of Applied Sciences, 53757 Sankt Augustin, Germany; argang.ghadiri@h-brs.de (A.G.); usha.singh@h-brs.de (U.S.); theo.peters@h-brs.de (T.P.); 2 Federal Institute for Occupational Safety and Health Dresden, 01099 Dresden, Germany; wendsche.johannes@baua.bund.de; 3 Institute of Movement and Neuroscience, German Sport University Cologne, 50933 Cologne, Germany; schneider@dshs-koeln.de

**Keywords:** experimental design, work break, performance measure, physiological measure, self-report measure, rest break, pause

## Abstract

The number of studies on work breaks and the importance of this subject is growing rapidly, with research showing that work breaks increase employees’ wellbeing and performance and workplace safety. However, comparing the results of work break research is difficult since the study designs and methods are heterogeneous and there is no standard theoretical model for work breaks. Based on a systematic literature search, this scoping review included a total of 93 studies on experimental work break research conducted over the last 30 years. This scoping review provides a first structured evaluation regarding the underlying theoretical framework, the variables investigated, and the measurement methods applied. Studies using a combination of measurement methods from the categories “self-report measures,” “performance measures,” and “physiological measures” are most common and to be preferred in work break research. This overview supplies important information for ergonomics researchers allowing them to design work break studies with a more structured and stronger theory-based approach. A standard theoretical model for work breaks is needed in order to further increase the comparability of studies in the field of experimental work break research in the future.

## 1. Introduction

From a social and economic perspective, companies are becoming increasingly aware of their responsibility for employees’ health. As a consequence, the corporate field has shown a rising interest in concepts of “corporate health management” and in a variety of offers related to health benefits for employees [1]. In the organizational setting, work breaks are considered to positively affect employees’ physical and cognitive performance, well-being, and health. 

The rationale behind work breaks was initially set in working conditions, prevalent decades ago. In times when work was generally associated with strenuous physical activities, employees became tired after a certain time, resulting in more variable or even declining task performance with time on task. They needed a rest to physically recover, often combining this rest with food intake. Although work break scheduling and regulations at that time were subjective and not backed by biological measurements [2], numerous countries passed at least some minimal standards for daily within-shift work breaks, as were passed for Europe [3]. However, in the meantime, despite a significant change in workplaces and their psychophysical demands, work break regulations and behavior have basically remained the same. 

Earlier research on work breaks focused on performance and productivity [4] and the relation to physical aspects [5] or on incidents and injuries (e.g., [6]). Over time, especially once computers were introduced into office settings and even more because of the change of computer work from less repetitive to more complex [5], research on work breaks changed and mental/psychological strains became a higher attention in work break research [5,7,8].

Investigations into work break research have been conducted in numerous study settings and with varying focuses. Research questioned or focused on either duration, timing, frequency or activity, or a combination of these aspects. Furthermore, there was diversity among the scientific disciplines interacting on work break research (e.g., ergonomics, psychology, work science, medicine) and the investigated variables (e.g., performance, productivity, stress, fatigue, mood, numerous physiological variables). This variety meant that study designs and measurement methods used in work break research were heterogeneous. As a result, there is currently a large number of measurement methods and variables for investigating physiological, psychological, and performance parameters and, unfortunately, with little standardization [9,10]. This is the natural consequence of variation in job design factors across work contexts, with the corresponding need to vary associated measures accordingly in relation to specific design factors.

Comparing work break studies is also difficult because the term “work break” has not been uniformly defined and there are so many different types. Work breaks are described with terms such as rest break, rest, lunch break, coffee break, pauses or break meaning the same or, sometimes, specific types of a work interruption. Wendsche and Lohmann-Haislah [11] differentiated between mandatory, optional, or supplementary work breaks and their length and reasons and between regulations in different countries. Mandatory work breaks can be binding for companies because of legislation or for employees because of company regulations. Work breaks can be planned by the company or the employees themselves. Furthermore, they can be triggered by operational procedures such as waiting time or production stops. Work breaks and work break behavior are often strongly linked to regulations in the different countries or even within industries, which can cause difficulties when work break behavior is under employee control [12], potentially leading to conflicts with the legislative regulations. Work breaks can also be distinguished by their length, lasting from a few seconds (micro break) to a few hours (siesta), by their content (e.g., coffee break, toilet break, lunch break), or by their intensity divided into active (e.g., stretching, walking, yoga) and passive (e.g., napping, relaxation) breaks. In our understanding, the term “work break” can be used as a general term to describe any intended interruption of work, regardless of length (e.g., micro break) or content (e.g., lunch break).

The effect of work breaks is multidimensional as they have an influence on physiological and psychological fatigue and strain, on mood, productivity, and social aspects, to name but a few. For example, if employees tire, they are generally less productive; a negative performance effect they can compensate for by working harder, which will lead to greater physiological or psychological strain. To capture these multidimensional effects, it makes sense to include methods from different measurement aspects. O’Donnell and Eggemeier [13] named five criteria for method selection that can also be transferred to work break research: sensitivity, diagnosticity, intrusiveness, implementation requirements, and acceptance by the operator. In particular, the aspects of sensitivity and diagnosticity are decisive aspects in the choice of suitable measurement methods. De Waard [14] also developed O’Donnell and Eggemeier’s [13] workload assessment methodology to a three-fold division of variables: self-report measures, performance measures, and physiological measures. The division into three categories is well suited for structuring effects in work break research.

Comparing work break studies is difficult as there is no theoretical model specialized on work breaks. As long as there is no independent theoretical model on the effect of work breaks, it makes sense to use as a basis the general models that take into account aspects of work breaks. Theoretical models that have already been used in the context of work breaks are the effort-recovery model by Meijman and Mulder (1998) (e.g., [15,16]) and the conservation of resources theory by Hobfoll (1989) (e.g., [17,18,19]).

The effort-recovery model [20] describes the positive development of recovery over a period of time when work-related demands no longer continue. Work breaks have an influence on the recovery of load reactions. Work break research can follow this theory by investigating forms of improving the recovery process. The conservation of resources theory [21] describes the stress-related loss of resources and predicts stress outcomes, e.g., in organizational settings. Researchers use this theory as a basis since work breaks influence stress processes and resource gain.

The aims of this review are: (1) To structure previously published intervention studies in work break research and (2) to evaluate them regarding their theoretical and methodical constructs. This review therefore provides a first structured overview of intervention studies on work breaks, their underlying theoretical models, and their measurement methods. This overview is important for ergonomics researchers when designing future work break studies and for extending theory-based knowledge of work breaks. It can help to maximize the positive potential of work breaks. Moreover, our review also intends to uncover possible methodological causes that may have led to inconsistent findings in earlier work break research [8,9,10,11].

## 2. Materials and Methods

To describe and summarize the variety of applied measurement methods in work break research, we conducted a scoping review based on the five stages described by Arksey and O’Malley [22]: (1) Identifying the research question, (2) identifying relevant studies, (3) study selection, (4) charting the data, and (5) collating, summarizing, and reporting the results. 

### 2.1. Stage 1: Identifying the Research Question

The scoping review was led by the following research questions: (a) Which measurement methods are used in work break research to investigate certain variables? (b) Are there any typical theoretical frameworks or hypotheses for work break research? and (c) Is it possible to standardize the future work break research?

Although we are aware of the problem of defining the term “work break,” we wanted to give an overview of applied measurement methods and so did not restrict the identified studies by the varying definitions of “work breaks,” accepting a wide definition. We did select the studies for further evaluation according to their intention to investigate work breaks in occupational settings or to give advice on occupational work design.

### 2.2. Stage 2: Identifying Relevant Studies

We started with a systematic review. To identify published studies on work breaks, electronic literature databases that are relevant for potential research in ergonomics, psychology, work science and management, and medicine were searched (PubMed, Web of Science, ScienceDirect), using a publication date from 1989 onward (last 30 years). The period of 30 years was chosen since at that time computers changed workplaces and working structures significantly. The three databases were searched in October 2017, and the search was updated in June 2019. Publications in languages other than English, review articles, book chapters, and conference proceedings were excluded from the methods’ evaluation.

Some work break studies have collected their data exclusively through questionnaires, diaries, or surveys. Those studies (e.g., [19,23,24]), which come close to a field study in the true sense, have a high value in understanding work breaks. However, because of the focus of this scoping review, studies without manipulation of either work break regimens or content were not included in the review.

For the literature search, we created a specific search string with the following main terms: (’work break*’ OR ‘break*’ OR ‘pause*’ OR ‘rest break*’ OR ‘lunch break*’) AND (‘work’ OR ‘job’ OR ‘occupation*’ OR ‘employ*’).

Additionally, a reference list search was conducted, especially for the systematic reviews and literature reviews described above, to ensure the inclusion of all studies (from 1989 onward) that other researchers had identified as relevant.

A hand-search of key ergonomics journals was conducted to find possibly missed articles from the database searches and references list searches. This search is recommended since electronic databases can be incomplete or outdated or because abstract services can vary in scope, indexing, and depth of information [22].

**Figure 1:**
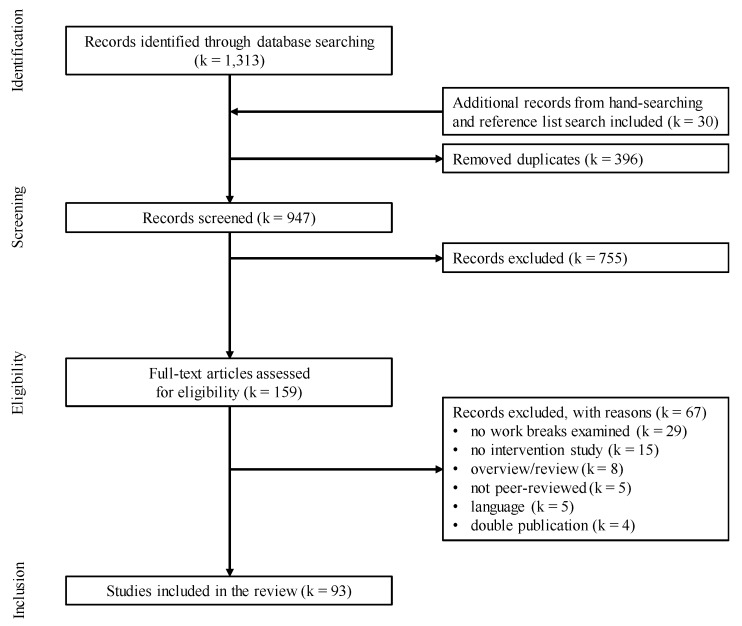
Flow chart on study selection.

The selection procedure and the outcomes are shown in Figure 1.

### 2.3. Stage 3: Study Selection

The search strategy identified numerous studies that do not focus on work breaks. Since the line of this scoping review was to identify as many measurement methods as possible, particularly strict exclusion criteria for different study designs were not necessary. Nevertheless, a few criteria had to be defined for a better understanding of the research field and to meet certain quality criteria. To be suitable, a study had to (1) investigate intra-shift work breaks, work break regimens, or work break activities, (2) be an intervention study (manipulating at least one aspect of (1)), (3) be peer-reviewed, (4) be in the English language, and (5) be published in full-text. Further quality criteria were not used.

The database search resulted in 1313 records. By excluding duplicates (k = 396) and adding further records from the reference list and hand searches (k = 30), a total of 947 records were screened by title and abstract. Of those records, 755 were discarded mostly because of not investigating on work breaks, resulting in 159 undergoing a full-text screening for suitability. The full-text screening resulted in 93 potentially eligible studies, which were finally included in the review (see Figure 1 for a flow chart).

### 2.4. Stage 4: Charting the Data

The key information of the selected studies was charted in line with the description by Arksey and O’Malley [22]. Comparable to a narrative review, the charted data were comprehensive and non-rating. The data (see Table A1) recorded the main information, such as general information (citation), study key data, investigated variables, and applied measurement methods. 

### 2.5. Stage 5: Collating, Summarizing, and Reporting the Results

In the next step, the data were transferred into a structure that could reflect the special features of work break research. For this purpose, a separate search was performed to find a suitable basic structure for the examined variables. The structure was then adapted to work break research (see Structure of Variables in Results). In the next step, the first author developed suitable variable-subcategories by viewing and clustering the variables examined by the studies. Afterward, three tables were created for structuring the variables, the variable-subcategories, and the applied measurement methods. One author performed data extraction. A second author checked this extraction for the listed measurement methods. Articles that did not define the applied methods clearly were excluded.

Unlike a systematic review, a scoping review does not synthesize evidence or summarize findings. Thus, this scoping review did not seek to assess quality of evidence and therefore does not determine the studies’ robustness or generalizability [22]; it describes the applied methods.

## 3. Results

Table A1 gives an overview of the characteristics of the studies. The following results of the research into measurement methods in work break analysis consists of methods from 93 publications from five continents: America (k = 36), Europe (k = 33), Asia (k = 17), Australia (k = 6), and Africa (k = 1). 55% of the studies were conducted in laboratories and 45% in the field. The review shows an increasing number of studies over the past 30 years, (1989–1998: k = 22; 1999–2008: k = 23; 2009–2019: k = 48). The examined activities can be divided into predominantly mental activities (67%) and predominantly physical activities (33%) including hospital activities (nursing, surgery), whereby many physical activities are of course also mentally demanding (e.g., concentration in surgery) or emotionally demanding (e.g., nursing). The studies were not dominated by specific industries or job titles. They investigated workplaces that can mainly be described as desk-based computer workstations where the participants worked during the studies. About 56% of the studies investigated different break regimens, whereas 44% of the studies focused on activity during work breaks. Two studies [25,26] investigated both break regimen and break activity; they were assigned to the break regimen for further evaluation as they focused on that topic. Almost half the studies (49%) were exploratory, without formulating a hypothesis. A total of 13 studies (14%) used a theoretical model as a study design basis. Of these 13 studies, only one theory (resource theory: [27,28]) occurred more than once. Furthermore, we found that within three decades the focus of work break research seems to be shifting from the United States to Europe and Asia (Table 1) and that the proportion of hypothesis- and theory-based studies has increased in the past decade (Table 2).

**Figure 2:**
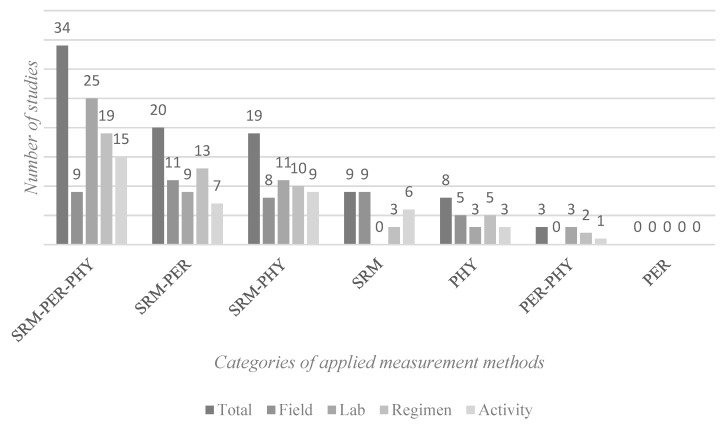
Method combinations in different study settings. Abbreviations: SRM = self-reported measures, PER = performance measures, PHY = physiological measures.

As described earlier, measurement methods can be divided into three groups: self-report measures (SRM), performance measures (PER), and physiological measures (PHY) [14]. SRM was used most frequently in 88% of the studies. PER was used in 61% of the studies, and PHY in 69%. Most of the studies (82%) used measurement methods from two or even all the categories. The combination of the applied measurement methods is shown in Figure 2. A combination of methods from all three categories can be found in more than a third of the studies (37%), followed by the combination of SRM and PER (22%) and SRM and PHY (20%). In field studies, the combination SRM and PER dominates (26%), and in laboratory studies, the SMR, PER, and PHY combination dominates (49%). The combination of SMR, PER, and PHY is most frequently used in the study of break regimens (37%) as well as for break activities (37%). Figure 2 gives an overview of the distribution of the method combinations.

De Waard’s [14] division into three methodological categories was further classified depending on the study situation. The structure thus provides an overview of the relevance of individual variables and measurement methods. Important for this review is that many of the studies used standardized questionnaires. However, these questionnaires are not always free of overlaps and the variables were investigated using different methods. The studies often only used certain items of a specific questionnaire to examine a variable.

### 3.1. Self-Report Measures

**Figure 3:**
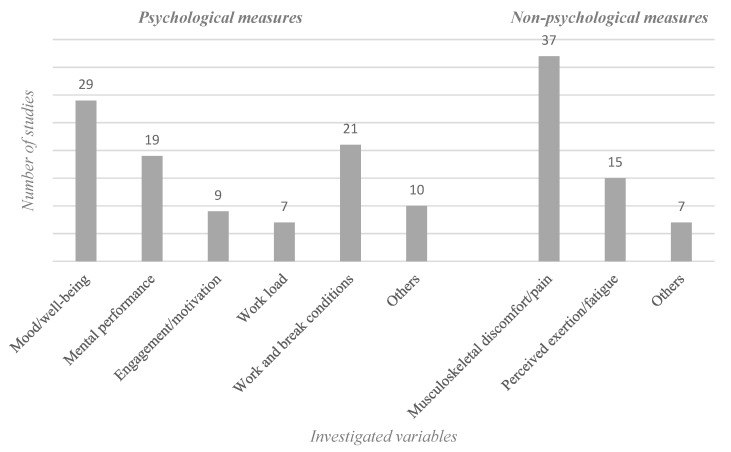
Self-reported measures and the subcategories psychological measures and non-psychological measures. Note: Number of studies investigating on certain variables by using at least one of psychological or non-psychological measures.

Self-report measures (see Figure 3), often indicated as subjective measures [14], were described by means of standardized or self-designed questionnaires (SDQ) and are the most popular assessment form in work break research. Within the self-report measures category, psychological and non-psychological measures can be distinguished. Within the psychological measures, the subdivisions mood/well-being, mental performance, engagement/motivation, workload, and work and break conditions are evident. Within the non-psychological measures, the two subdivisions musculoskeletal discomfort/pain and perceived exertion/fatigue arise.

#### 3.1.1. Psychological Measures

*Mood and Well-being***.** The variable mood/well-being is a central object of investigation in work break research. To measure this state of mind, the *Profile of Mood States* (POMS; [29]) was most commonly applied. In addition, numerous studies used self-designed questionnaires, often method-based on the Visual Analogue Scale (VAS; first described by Hayes and Paterson [30]), or selecting single scales or items (e.g., rating stress or sleep) from standardized questionnaires.

*Mental performance*. This variable evaluates cognitive performance in terms of alertness, attention, and concentration. Contrary to this positive state of mind in which personal best possible performance can be achieved, there is sleepiness (usually short term) or fatigue (often a longer lasting state). The Stanford Sleepiness Scale (SSS; [31]) and the Karolinska Sleepiness Scale (KSS; [32]) were the main instruments to examine these mental states.

*Engagement and Motivation.* The engagement and motivation to accomplish a task are also of interest when considering the effects of a work break and have been investigated using various methods.

*Workload.* This variable is used to determine a subject’s perceived workload. The predominant examination method was the NASA Task Load Index (NASA-TLX; [33]). 

*Work and break conditions.* The category work and break conditions examines aspects of the workplace design and framework conditions and the associated work break design. Motivation for and continuity in practicing the investigated (and mostly newly introduced) work break or work break design also belong to this category. Usually, self-designed questionnaires or diaries were applied here. In evaluating the work breaks, self-designed questionnaires asked for aspects such as personal preferences and popularity as well as possible barriers. Since these self-designed questionnaires are usually not published and evaluated, they are not discussed in this review.

*Others.* In addition to these five categories and the related methods, numerous other self-reported methods (e.g., on quality of life or detachment) have been applied in work break studies. These are diverse and have only rarely been used in work break research. They are therefore not discussed in depth in this review.

#### 3.1.2. Non-Psychological Measures

*Musculoskeletal discomfort/pain.* In the domain of non-psychological assessments, complaints about muscular discomfort or even pain were the most frequently studied variables.

Most of the studies used a Visual Analogue Scale, based on the Borg category ratio scaling (e.g., Borg CR10; [34,35,36]), to evaluate the general physical condition or, more often, individual body parts. The neck and back were the most frequently examined.

Other tools are a Body Part Diagram/Discomfort Scale developed by Corlett and Bishop [37] and a Visual Analogue Discomfort scale [38].

*Perceived exertion/fatigue.* Perceived exertion was another variable used to assess the physical effects of work breaks. For this purpose, the Borg Rating of Perceived Exertion Scale (Borg RPE scale; [35,39]) has been applied almost universally. It is used to estimate effort and exertion during physical work, among other variables. 

*Others.* Of the other investigated variables, physical fatigue and physical activity should be highlighted. Physical fatigue was measured differently and was surveyed or determined by physical strength exercises. Physical activity, on the other hand, was usually determined using the International Physical Activity Questionnaire (IPAQ; [40]).

### 3.2. Performance Measures

**Figure 4:**
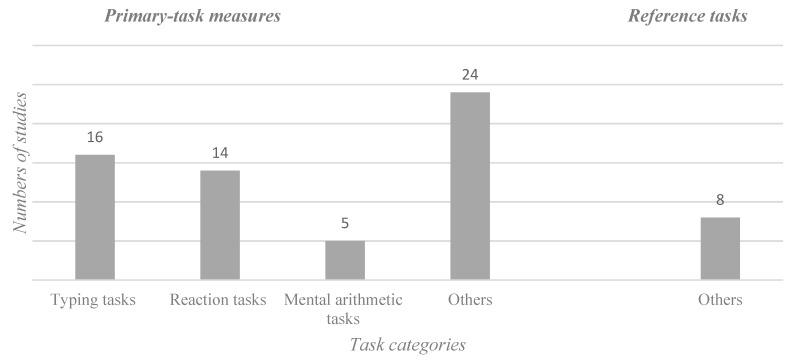
Performance measures and the subcategories primary-task measures and reference tasks. Note: Number of studies investigating different task categories.

According to de Waard [14], performance measures can be grouped into primary-task measures, secondary-task measures, and reference tasks. For work break research, that grouping can be reduced to primary tasks and reference tasks (Figure 4).

Primary tasks were utilized to measure the overall effectiveness. Reference tasks were performed before and after the work break to investigate performance changes through a specific work break regimen or work break activity.

#### 3.2.1. Primary Tasks

In field studies, primary tasks were task specific because of the specificity of the investigated workplace. During quasi-experimental field studies, primary tasks were implemented in the working process and served as a measurable and comparable performance indicator. In laboratory studies, the primary tasks were structured into a few, common tasks. These tasks were often self-designed but aimed at the same measures, such as motor performance, speed of performance, reaction time, accuracy, and number of errors.

Tasks used to measure performance can be divided into typing tasks (keystrokes, error rate, correction rate, number of words typed), reaction tasks (reaction time, accuracy, choice reaction time, auditory reaction time task), and mental arithmetic tasks (mathematical calculation task).

#### 3.2.2. Reference Tasks

Reference tasks mainly serve to check for trend effects and are performed under evaluation before and after the task [14], or for work break research before and after the break. Common tasks were memory tasks, concentration tasks, and reaction tasks. The only standardized tasks that was used more than once as a reference task was the *d2 Test of Attention* (d2; [41]; d2-R; [42]).

### 3.3. Physiological Measures

**Figure 5:**
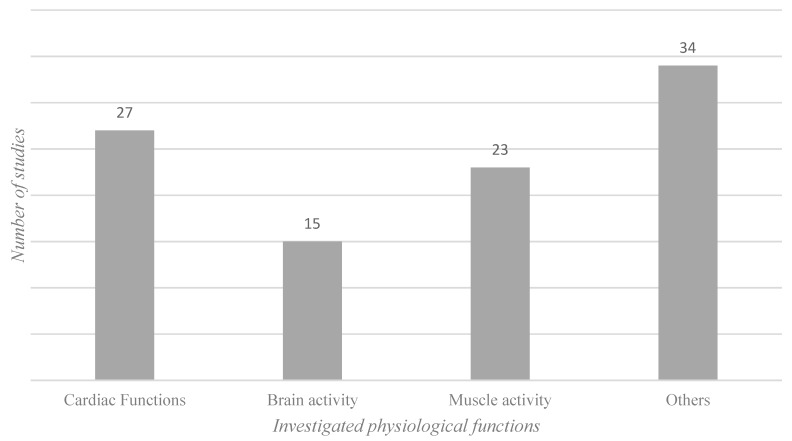
Physiological measures. Note: Number of studies investigating different physiological functions.

Within the wide range of physiological measurement methods, four methods stand out because of their frequency (Figure 5). The categories cardiac functions, brain activity, and muscle activity are therefore described in more detail below. Other less frequently applied physiological measurement methods are mentioned in the Others category.

#### 3.3.1. Cardiac Functions

The cardiac function can be described as the heart’s ability to fulfill the metabolic demands of the body. As a result of increased metabolic need, e.g., caused by stress or physical exertion, oxygen demand will increase. To fulfill the oxygen demands, the body can increase the respiratory rate or increase heart rate. The electrocardiogram (ECG) can represent the cardiac function by providing information about the electrical activity conduction through the heart [43].

To assess physiological function (and indirect through this, sometimes also mental load, e.g., stress), numerous studies in work break research have measured heart rate and heart rate variability (also described as R-R interval or interbeat interval).

#### 3.3.2. Brain Activity

The activity of the brain is conventionally measured using the encephalogram (EEG), which displays oscillations in several, functionally relevant frequency bands [44]. EEG measurements are conducted using one of two methods: Spontaneous EEG, when the neural oscillations of the brain waves are considered, and the event-related potentials (ERP), when the reaction of the EEG after an evoked stimulus is studied. ERPs are often used to determine the reaction time. Functional magnetic resonance imaging (fMRI) is another tool to measure brain activity applied in work break research. In contrast to the EEG, the fMRI shows regional, time-varying changes in the brain activity but is more complex to use because of the clinical scanner involved.

#### 3.3.3. Muscle Activity

Another physiological measurement of interest is the activity or inactivity of certain muscles. Muscle activity is investigated in particular in connection with physical labor, monotonous or one-sided movements, or sedentary work behavior, with the main focus on neck and shoulder. In particular, the trapezius muscle is used as a standard to measure the shoulder-neck load in occupational or ergonomics studies [45]. The dominant measurement tool is electromyography (EMG) or, more precisely, surface electromyography (sEMG). In work break research, this non-invasive method is usually used.

#### 3.3.4. Others

Further methods in work break research to determine physical functions or the changes of physical parameters because of a certain break regimen or work break activity are eye functions (through electrooculography), spinal shrinkage (through a stadiometer), rest/activity (through actigraphy), sleep, electrodermal activity, temperature, and hormone levels.

## 4. Discussion

This scoping review examined the work break research conducted over the past 30 years (1989 to 2019). We reviewed (1) the development and domains of research in this area, (2) the theoretical rationale behind this research, and (3) the methodological framework used. Using data from 93 studies, this review therefore goes beyond the existing work and is the first review to focus on the theoretical and methodological approaches.

First, an increasing interest in the effect of work breaks and thus an increasing number of studies on work breaks can be clearly observed for the period investigated. In 2009, Trougakos and Hideg [46] identified ergonomics literature as the primary domain investigating within-day work breaks. In 2019, ergonomics literature is still the main publication channel for work break research but its dominance has changed, and research in the area has now been extended to “medicine,” “neurology,” “physiology,” and others. The reviewed studies come from various countries, with the industrial nations clearly dominating the research activities. The work areas examined in the studies are dominated by computer workstations. However, there are also some studies that investigate activities defined by mainly physical activity.

Second, the difficulty in comparing work break studies and their results could be explained by the heterogeneous approach taken in designing and realizing empirical studies. Nearly half of the studies were exploratory and only 14% of the studies were based on a theoretical model (e.g., resource theory). This is problematic as interpretation of study findings is limited, especially in this case of research where results have been often reported inconsistent across studies. Moreover, it seems that this lack of theory-driven approaches has developed into repeated investigations of rather simple research questions (e.g., Do work breaks reduce mental and physical strain? Which break activities reduce mental and physical strain?). In turn, current knowledge on mechanisms and moderators shaping effects of work breaks is sparse. Therefore, future work break research should be more theoretically guided to explain if, when, and why work breaks have any effects on certain outcomes.

Third, with regard to the issues discussed above, the measurement methods used varied greatly across the studies. The measurement methods were sorted according to three categories of measurement variables: “self-report measures,” “performance measures,” and “physiological measures.” The choice of variables to be investigated depended on the study design and the research question. Ideally, a study design considers variables from two or even all three categories [14].

To examine the multidimensional effects of a work break, using a number of measurement methods is reasonable and a large number of methods for measuring each individual variable are already available, especially in the case of self-reported measures. In most papers, the authors explained their method choice simply by referring to the method being used in previous work. Few studies provide an independent evaluation of the method, especially with regard to the sensitivity and diagnosticity, which are related to the individual study design. The methodological heterogeneity of the research limits a final evaluation of previous results in all relevant aspects of work break research such as performance, motivation, well-being, health, break regimen, break location, and break content [11]. 

A combination of measurement methods of self-report measures, performance measures, and physiological measures is a promising approach to determine interdependencies of the multidimensional effects [11,14] and has been repeatedly recommended for future research (e.g., [18,23]). The combination of measurement methods from the three measure categories will lead to better results. These methods should be evaluated regarding the five criteria by O’Donnell and Eggemeier [13] especially sensitivity and diagnosticity. Furthermore, the scientific community recommends describing the methods’ objectivity, validity, and reliability. A description of objectivity, validity, and reliability (e.g., [47,48]) was given on 11% of the reviewed studies. Especially in studies with a small number of test persons, effect sizes should be reported (e.g., [49,50]). 

The sample selection and sample planning are other critical points. As in many other areas of research, rest break research often uses students as participants in lab studies. In studies that investigate aspects such as stress experience, physical discomfort, and compensatory capacity, this group of young and, in general, healthier people is not necessarily transferable to the work situation and the average employee. Sample planning can only be found sporadically in the studies investigated (e.g., [51]), but could help to improve results in terms of practical relevance in the future.

A central role in evaluating measurement results in work break research is certainly played by the individual level of demands. The individual course of demands and the individual course of recovery from these demands still offer investigative potential and would allow work break effects to be evaluated from the process perspective. In particular, for a detailed assessment of the performance and recovery processes, a distinction must be made between an investigation of the demands and recovery at one point in time or over a certain period of time.

We would like to note that the results of our review are limited by scope of literature search (e.g., selection of electronic databases, search string, inclusion and exclusion criteria). However, we think that the potential of a publication bias is low here as we used databased literature search, inspection of references, and hand-search of journals in combination which resulted in a much higher number of included studies than in other current reviews on work breaks [8,9,10,11].

## 5. Conclusions

Work breaks are an important measure to offer employees time to immediately recover from work-related consequences of strain after high work demands and, in turn, to improve their mental and physical wellbeing, their performance, and workplace safety.

In our review we set out to structure and evaluate the experimental work break research from 1989 to 2019 (k = 93 studies), since there are manifold ways of analyzing the effects of work breaks regarding study designs and measurement methods.

Most of the examined studies were exploratory, thus, missing a theoretical foundation. Moreover, during the last 30 years most researchers applied previously used measurement methods without evaluating them for their own studies; neither regarding criteria as sensitivity and diagnosticity nor regarding the methods’ objectivity, validity, and reliability.

In sum, in future studies it would be desirable to develop a theoretical model that is focused on the particular and various effects of work breaks and can serve as a standard model for future work break studies [52]. Furthermore, variables measured by methods out of all three categories (“self-report measures,” “performance measures,” and “physiological measures”) should be considered in future study designs. 

## Figures and Tables

**Table 1:** Change in countries of origin in the last three decades.

Continent	k	1989–1998	1999–2008	2009–2019
America	36	14	9	13
Europe	33	7	8	18
Asia	17	1	4	12
Australia	6	0	2	4
Africa	1	0	0	1
Total	93	22	23	48

**Table 2:** Distribution of selected study contents over the last three decades.

Study Design	k	1989–1998	1999–2008	2009–2019
Lab study	51	17	7	27
Field study	42	5	16	21
Mental work task	62	15	10	37
Physical work task	31	7	13	11
Hypothesis-based	46	6	8	32
Theory-based	13	0	0	13

## Appendix A

**Table A1:** Characteristics and categories of applied measurement methods of included studies. Abbreviations: SRM = self-reported measures, PER = performance measures, PHY = physiological measures.

											Applied Measurement Methods		
Author	Year	Country	Site	Work/Task	Sample Size	Days	Time-on-Task	Object	Hypothesis	Theory/Model	SRM	PER	PHY
**Arrabito et al. [53]**	**2011**	Canada	Lab	Mental	98	1	40 min	Activity	no	no	yes	yes	
**Arrabito et al. [54]**	**2015**	Canada	Lab	Mental	150	1	40 min	Activity	yes	yes	yes	yes	
**Balci and Aghazadeh [55,56,57]**	**2000 (2003, 2004)**	USA	Lab	Mental	10	1	120 min	Regimen	no	no	yes	yes	yes
**Beynon and Reilly [58]**	**2001**	UK	Lab	Physical	10	2	240 min	Activity	yes	no	yes		yes
**Beynon et al. [59]**	**2000**	UK	Lab	Physical	8	2	full working day	Regimen	yes	no	yes		yes
**Boucsein and Thum [5,60]**	**1995 (1997)**	Germany	Field	Mental	11	2	full working day	Regimen	no	no	yes		yes
**Brown et al. [51]**	**2014**	UK	Field	Mental	73	56	full working day	Activity	yes	no	yes		yes
**Byström et al. [61]**	**1991**	Sweden	Lab	Physical	8	1	3 min	Activity	no	no	yes	yes	yes
**Chaikumarn et al. [62]**	**2018**	Thailand	Lab	Mental	30	1	60 min	Activity	yes	no		yes	yes
**Chakrabarty et al. [63]**	**2016**	India	Field	Physical	400	120	360 min	Regimen	yes	no	yes		
**Chavaillaz et al. [64]**	**2019**	Switzerland	Lab	Mental	72	1	60 min	Regimen	yes	yes	yes	yes	
**Christensen et al. [65]**	**2000**	Denmark	Field	Physical	48	1	full working day	Regimen	yes	no			yes
**Coburn et al. [66]**	**2006**	Germany	Field	Physical	30	2	450 min	Regimen	yes	no	yes	yes	
**Constable et al. [67]**	**1994**	USA	Lab	Physical	8	1	180 min	Activity	yes	no			yes
**Cordoza et al. [68]**	**2018**	USA	Field	Physical	29	84	full working day	Activity	no	no	yes		
**Crenshaw et al. [69]**	**2006**	Sweden	Lab	Mental	15	2	60 min	Activity	yes	no	yes	yes	yes
**Dababneh et al. [70]**	**2001**	USA	Field	Physical	30	36	full working day	Regimen	no	no	yes	yes	
**Davis and Kotowski [71]**	**2014**	USA	Field	Mental	37	40	full working day	Regimen	no	no	yes	yes	yes
**Davy and Göbel [25]**	**2013**	Germany	Lab	Physical	24	3	full night shift	Regimen	no	no	yes	yes	yes
**de Looze et al. [72]**	**2010**	The Netherlands	Field	Physical	14	3	440 min	Regimen	no	no	yes	yes	
**Dorion and Darveau [73]**	**2013**	Canada	Field	Physical	16	3	full working day	Regimen	yes	no	yes	yes	yes
**Engelmann et al. [74]**	**2011**	Germany	Field	Physical	7	1	full working day	Regimen	yes	no	yes	yes	yes
**Faucett et al. [75]**	**2007**	USA	Field	Physical	66/32	2/6	480 min	Regimen	no	no	yes	yes	
**Frey et al. [76]**	**2002**	Austria	Field	Physical	11	2	full 24h shift	Activity	no	no	yes	yes	yes
**Galinsky et al. [77]**	**2000**	USA	Field	Physical	42	60	full working day	Regimen	no	no	yes	yes	
**Galinsky et al. [78]**	**2007**	USA	Field	Mental	51	56	full working day	Regimen	no	no	yes	yes	
**Garcia et al. [79]**	**2018**	Switzerland	Lab	Physical	30	2	300 min	Regimen	yes	no	yes		yes
**Genaidy and al-Rayes [80]**	**1993**	USA	Lab	Physical	5/8	2	240 min	Regimen	no	no	yes	yes	yes
**Genaidy et al. [81]**	**1995**	USA	Field	Physical	28	28	full working day	Regimen	no	no	yes		
**Hayashi et al. [82]**	**2004**	Japan	Field	Mental	10	2	120 min	Activity	no	no	yes	yes	yes
**Helander and Quance [83]**	**1990**	USA	Lab	Mental	7	4	200 min	Regimen	yes	no			yes
**Henning et al. [84]**	**1989**	USA	Lab	Mental	20	2	120 min	Regimen	yes	no	yes	yes	yes
**Henning et al. [85]**	**1993**	USA	Lab	Mental	38	1	48 min	Regimen	no	no	yes	yes	
**Henning et al. [86]**	**1994a**	USA	Lab	Mental	31	1	65 min	Regimen	no	no	yes	yes	yes
**Henning et al. [87]**	**1994b**	USA	Lab	Mental	38	1	48 min	Regimen	no	no	yes	yes	yes
**Henning et al. [88]**	**1995**	USA	Lab	Mental	30	1	55 min	Regimen	no	no	yes	yes	yes
**Henning et al. [89]**	**1996**	USA	Lab	Mental	31/30	1	60 min	Regimen	no	no	yes	yes	yes
**Henning et al. [26]**	**1997a**	USA	Field	Mental	73/19	28/42	full working day	Regimen	yes	no	yes	yes	
**Henning et al. [90]**	**1997b**	USA	Lab	Mental	60	1	46 min	Regimen	no	no	yes	yes	yes
**Hirose and Nagasaka [91]**	**2003**	Japan	Lab	Mental	8	1	100 min	Activity	no	no	yes	yes	yes
**Hofer-Tinguely et al. [92]**	**2005**	Switzerland	Lab	Physical	50	1	60 min	Activity	no	no	yes	yes	yes
**Howard et al. [93]**	**2010**	Australia	Lab	Mental	8	3	full night shift	Regimen	no	no	yes	yes	yes
**Irmak et al. [94]**	**2012**	Turkey	Field	Mental	39	70	full working day	Regimen	no	no	yes		
**Januario et al. [95]**	**2018**	Brazil	Lab	Physical	18	1	40 min	Activity	yes	no	yes		yes
**Kakarot et al. [96]**	**2012**	Germany	Lab	Physical	29	2	420 min	Regimen	yes	no	yes		yes
**Karwowski et al. [97]**	**1994**	USA	Lab	Mental	12	12	240 min	Regimen	yes	no	yes		yes
**Kennedy and Ball [98]**	**2007**	Australia	Field	Mental	75	28	full working day	Activity	yes	no	yes		
**Kietrys et al. [99]**	**2007**	USA	Field	Mental	72	28	full working day	Activity	no	no	yes		
**Kopardekar and Mital [100]**	**1994**	USA	Lab	Mental	8	1	120 min	Regimen	no	no	yes	yes	
**Krajewski et al. [101]**	**2011**	Germany	Field	Mental	14	168	full working day	Activity	yes	yes			yes
**Lacaze et al. [102]**	**2010**	Brazil	Field	Mental	64	40	full working day	Activity	no	no	yes		
**Larsen et al. [103]**	**2009**	Denmark	Field	Mental	18	1	30 min	Activity	yes	no	yes	yes	yes
**Lim and Kwok [104]**	**2016**	Singapore	Lab	Mental	71	1	60 min	Regimen	yes	yes	yes	yes	
**Lim et al. [105]**	**2013**	Singapore	Lab	Mental	31	2	65 min	Regimen	yes	no		yes	yes
**Lim et al. [106]**	**2016**	Singapore	Lab	Mental	27	2	1 min	Regimen	yes	no		yes	yes
**Mailey et al. [107]**	**2016**	USA	Field	Mental	49	56	full working day	Regimen	no	no	yes		yes
**Mathiassen & Winkel [108]**	**1996**	Sweden	Lab	Physical	8	7	240–360 min	Activity	no	no	yes		yes
**Mathiassen et al. [109]**	**2014**	Sweden	Lab	Mental	18	3	42 min	Regimen	no	yes	yes	yes	yes
**McLean et al. [110]**	**2001**	Canada	Field	Mental	15	30	full working day	Regimen	yes	no	yes	yes	yes
**Miller and Fathallah [111]**	**2006**	USA	Field	Physical	14	1	55 min	Regimen	no	no			yes
**Mitra et al. [112]**	**2008**	Australia	Field	Physical	233	28	full working day	Regimen	no	no	yes	yes	
**Mohamed and Radwan [113]**	**2017**	Saudi Arabia	Lab	Mental	45	1	60 min	Regimen	no	no			yes
**Nakphet et al. [114]**	**2014**	Thailand	Lab	Mental	30	1	60 min	Activity	no	no	yes	yes	yes
**Neri et al. [115]**	**2002**	USA	Lab	Mental	28	1	360 min	Regimen	yes	no	yes		yes
**O’Donnell et al. [116]**	**2015**	Australia	Lab	Mental	60	1	48 min	Regimen	yes	yes	yes	yes	yes
**Oriyama et al. [117]**	**2014**	Japan	Field	Physical	15	1	full night shift	Regimen	no	no	yes		yes
**Rahman et al. [118]**	**2018**	Malaysia	Field	Physical	15	1	160 min	Regimen	no	no			yes
**Reyner & Horne [119]**	**1997**	UK	Lab	Mental	12	1	180 min	Activity	no	no	yes	yes	yes
**Ross et al. [120]**	**2014**	New Zealand	Lab	Mental	40	1	40 min	Activity	yes	yes	yes	yes	
**Rupp et al. [121]**	**2017**	USA	Lab	Mental	65	1	15 min	Activity	yes	yes	yes	yes	
**Samani et al. [122]**	**2009a**	Denmark	Lab	Mental	12	2	12 + 4 min	Activity	yes	yes	yes		yes
**Samani et al. [123]**	**2009b**	Denmark	Lab	Mental	12	1	40 min	Activity	yes	no	yes		yes
**Schnieder et al. [124]**	**2013**	Germany	Field	Mental	14	180	full working day	Activity	yes	no	yes	yes	
**Scholz et al. [50]**	**2018**	Germany	Lab	Mental	12	4	120 min	Activity	yes	no	yes	yes	yes
**Sheahan et al. [125]**	**2016**	Canada	Lab	Mental	20	4	60–70 min	Regimen	yes	no	yes	yes	yes
**Sianoja et al. [15]**	**2017**	Finland	Field	Mental	97	10	full working day	Activity	yes	yes	yes		
**Sommer et al. [126]**	**2013**	Germany	Lab	Mental	32	2	0	Activity	yes	no	yes	yes	yes
**Steinborn and Hystegge [127]**	**2016**	Germany	Lab	Mental	68	1	50 min	Activity	yes	yes	yes	yes	
**St-Onge et al. [128]**	**2017**	Canada	Lab	Mental	27	1	40 min	Activity	yes	no	yes	yes	yes
**Sun et al. [49]**	**2017**	Singapore	Lab	Mental	20	2	22 min	Regimen	yes	yes	yes	yes	yes
**Sundelin [129]**	**1993**	Sweden	Lab	Physical	12	1	120 min	Activity	no	no	yes		yes
**Sundelin and Hagberg [130]**	**1989**	Sweden	Field	Mental	12	1	90 min	Activity	no	no	yes		yes
**Takahashi et al. [131]**	**1998**	Japan	Lab	Mental	30	1	full working day	Regimen	yes	no	yes	yes	yes
**Takahashi et al. [132]**	**2004**	Japan	Field	Physical	8	21	full working day	Activity	no	no	yes	yes	yes
**Taylor et al. [133]**	**2013**	USA	Field	Mental	35	180/365	full working day	Activity	no	no	yes		
**Taylor et al. [47]**	**2016**	USA	Field	Mental	175	180	full working day	Activity	yes	no	yes		yes
**Tiwari and Gite [134]**	**2006**	India	Field	Physical	5	4	360 min	Regimen	no	no	yes		yes
**Torrente et al. [135]**	**2017**	Finland	Field	Mental	153	30	full working day	Activity	yes	yes			yes
**van den Heuvel et al. [136]**	**2003**	The Netherlands	Field	Mental	268	60	full working day	Activity	no	no	yes	yes	
**van Dieen and Oude Vrielink [137]**	**1998**	The Netherlands	Field	Physical	12	3	full working day	Regimen	no	no	yes		yes
**Wang and Pei [138]**	**2014**	China	Field	Mental	33	1	120–240 min	Regimen	no	no	yes	yes	
**Watling et al. [48]**	**2014**	Australia	Lab	Mental	20	1	180 min	Activity	yes	no	yes	yes	yes
**Wollseiffen et al. [139]**	**2015**	Germany	Field	Mental	50	1	full working day	Activity	yes	no	yes	yes	yes
